# Effects of fermented dried brewer's grain on performance, carcass characteristics, morphology of ileum and blood parameters in broiler chickens

**DOI:** 10.1016/j.psj.2025.105284

**Published:** 2025-05-14

**Authors:** Mohammad Khosravi, Behrouz Dastar, Omid Ashayerizadeh, Fariborz Khajali

**Affiliations:** a Department of Animal and Poultry Nutrition, Faculty of Animal Science, Gorgan University of Agricultural Sciences and Natural Resources, Shahid Beheshti Ave, Gorgan, Iran; b Department of Animal Science, Shahrekord University, Shahrekord, Iran

**Keywords:** Blood parameters, Broiler chicken, Dried brewer's barley, Fermentation, Ileum morphology

## Abstract

This experiment investigated the effects of fermentation on the chemical composition of dried brewer's grain (DBG) and its inclusion in broiler diets on performance, carcass traits, intestinal histology, and blood proteins. The DBG was fermented for 21 days using *Bacillus subtilis* and *Aspergillus oryzae*. A total of 360 male broilers (Ross 308) were fed with 10 % or 20 % DBG or fermented DBG (FDBG) for 42 days. Results revealed that fermentation could reduce crude fiber, pH, nitrogen-free extract, neutral detergent fiber, acid detergent fiber, and total bacterial count whereas it increased Lactobacillus count as well as fat, ash, calcium, and phosphorus contents (*P<0.01*). Broilers fed 10 % FDBG exhibited a similar performance to the control group. Serum levels of total protein and globulin were significantly higher in broilers that received 10 % FDBG compared to the control (*P<0.01*). However, feeding broilers with 20 % DBG or 20 % FDBG reduced weight gain and worsened the feed conversion ratio (*P* < 0.001). Moreover, broilers that received 20 % DBG had a shorter ileal villus height compared to the control group (*P=0.034*). These findings suggest that microbial fermentation could improve the nutritional quality of DBG, and the fermented product (FDBG) could be included in broiler diets up to 10 % without impacting performance.

## Introduction

The malting process is a controlled germination of barley by steeping seeds in water and crushing them under controlled temperature and relative humidity conditions (Lynch et al., 2016). Brewer's spent grain (BSG) is the remaining solid phase of the brewer in the malting process (Mussatto et al., 2006; Zeko Pivac, 2022). The BSG is mainly composed of seed coat-pericarp-husk layers, which contains an enormous content of cellulose, non-starch polysaccharides (NSPs), lignin, as well as protein and lipids. To some extent, the starchy endosperm and the aleurone layer may remain in BSG, depending on the efficiency of mashing (Lynch *et al*., 2016; Mussatto et al., 2006). A significant amount of silica, along with a substantial portion of polyphenolic components of barley grain may also be found in BSG (Macleod, 1979; Lynch *et al*., 2016). The most abundant by-product generated from the brewing process is BSG, accounting for 85 % of total by-products (Zeko-Pivac, 2022). Globally, the BSG production reaches approximately 39 million tons annually (Mussatto et al., 2006), posing a substantial disposal challenge for breweries, including those producing beer and non-alcoholic beverages (Canonico *et al*., 2024). It is estimated that about 20 kg of BSG is rendered per each liter of beer produced (Gupta *et al*., 2010). The nutritional value of the by-product (BSG) is high depending on plant maturity, processing method, and added ingredients during the beverage production process (Abd El-Hack *et al*., 2019).

BSG can be dried and fed to poultry as a feedstuff, known as dried brewer's grain (DBG), which contains 87 % dry matter, 22,1 % crude protein, 12.3 % crude fiber, 4.8 % ash, and 2485 kcal/kg metabolizable energy (Fasuyi *et al*., 2018). El-Hack *et al*. (2019) reported that DBG protein consisted of 4.8 % alanine, 4.1 % arginine, 5.9 % aspartic acid, 1.8 % cystine, 19.9 % glutamic acid, 3.4 % glycine, 1.8 % histidine, 4.2 % isoleucine, 8.2 % leucine, 3.1 % lysine, 1.5 % methionine, 5.3 % phenylalanine, 8.8 % proline, 4.0 % serine, 3.2 % threonine, 1.2 % tryptophan, 3.5 % tyrosine, and 4.8 % valine as a percent of protein content. Denstadli *et al*. (2010) examined dietary levels of DBG from 10 % to 50 % (replacing corn and soybean) in broiler chicken diets. Their findings showed that dietary levels of DBG above 20 % significantly reduced growth performance. Alabi *et al*. (2014) reported that dietary inclusion of DBG exceeding 25 % decreased growth performance in broiler chickens. The limiting factors in the use of DBG for broiler chickens are high concentrations of NSPs and tannins. Dietary fiber of DBGs contains 20-25 % hemicellulose, 12-25 % cellulose, and 10-28 % lignin (Lynch *et al*., 2016). Hemicellulose is the main component of DBG, which comprises approximately 40 % of the hemicellulose surface area and is composed of arabinoxylans (Forssell *et al*., 2008).

A major challenge in the use of DBG is the binding between protein and fiber components. Biological methods can be employed to enhance the nutritional value and reduce anti-nutritional compounds in this valuable feed (Tan *et al*., 2019). Solid-state fermentation (SSF) technique is a biological method for reducing anti-nutritional factors and increasing the bioavailability of feed. Because SSF does not require the use of solvents or chemicals and it does not produce toxic compounds, it is considered an environmentally friendly approach. Additionally, SSF is attractive to researchers, not only for its environmental benefits, but also for aligning with the principles of sustainable feed production, utilizing agricultural by-products and waste materials to create a cost-effective and resource-efficient livestock feed (Xu *et al*., 2012; Sugiharato *et al*., 2016). This method is also more efficient and effective than other methods (Sugiharto and Ranjitkar, 2019). Microbial fermentation can reduce the carbohydrate level (D-fructose, gluconic acid, maltose, d-mannitol, d-galactose, heptadecanoic acid) while increasing the concentration of amino acids, essential fatty, acids and antioxidants (Tan *et al*., 2019). These changes improve the nutritional value of DBG for broilers. Al-Khalaifah *et al*. (2020) used 10 % fermented DBG in broiler chicken diets. They reported improved weight gain, feed conversion ratio (FCR), and improved nutrient digestion by the over-expression of enzymes (proteases, lipases and amylases). Given the limited studies on the use of DBG in poultry diets, this study aimed to compare the chemical composition of raw and fermented DBG and to investigate the effects of using raw and fermented DBG (FDBG) levels on performance, carcass composition, morphology of ileum, and blood parameters in broiler chickens.

## Materials and methods

All methods used in this study were approved by the Animal Care and Use Committee of Gorgan University of Agricultural Sciences and Natural Resources, Gorgan, Iran, under protocol 477 N-10-11.

### Preparation of DBG and FDBG

A private non-alcoholic beer factory in Shahrekord Iran, provided BSG. The process of DBG went through three stages: steeping, germination, and drying. At the steeping stage, barley was cleaned, washed, disinfected and then put into a stainless-steel tank full of water from 5°C to 18°C for about 48 hours. Soaked grains were then moved into the germination room where the temperature was 15°C to 21°C, and humidity was over 70 %, preparing barley seeds to germinate after 6 to 7 days. During this process, the synthesis and activation of hydrolyzing enzymes in the aleurone and starchy endosperm, mainly amylases, proteases, and glucanases, were promoted. At the drying stage, malted barley grains were dried at 40–60°C to produce flavor components. In the brewing stage, malted barley underwent milling and mixed with water at temperatures gradually raised from 37 to 78°C, which could facilitate the enzymatic hydrolysis of the components of malt, mainly starch, proteins, and NSPs including mixed-ꞵ-glucans and arabinoxylans.

A sweet liquid, known as wort, was obtained from the enzymatic conversion stage. The unbreakable part of the malted barley grain was permitted to settle into a mash tun in a process known as lautering. The sweet wort was filtered through the mash tun. Subsequently, the filtered wort served as the medium for fermentation in beer production (Mussatto et al., 2006). Lynch *et al*. (2016) noted that the process of brewing selectively extracts only the required nutrients from the malt to produce the wort, which leaves washed, water-insoluble proteins and the cell wall residues of the husk, pericarp and seed coat within the spent grain behind. The BSG was dried for 36 hours at 45°C, and DBG was produced. The content of NSPs of barley and DBG was measured (Slominski and Campbell, 1990) and reported in Table 1.

**Table 1:** NSPs component of barley and DBG (as mg/g).

	Barley	DBG
Arabinose	20.24	23.97
Xylose	49.37	57.00
Mannose	3.85	2.11
Galactose	2.75	3.21
Glucose	79.14	57.72
Uronic acids	4.98	6.07
Total NSPs	160.28	150.07

DBG was inoculated at 33°C for 21 days with *Bacillus subtilis* (PTCC1156) bacteria and *Aspergillus oryzae* (PTCC5163) fungus (Ashayerizadeh et al., 2017) in a vacuum tank to produce FDBG. The details of the fermentation process were reported previously elsewhere (Ashayerizadeh *et al*., 2017). Finally, FDBG was dried at 45°C for 48 hours.

### Chemical composition and microbial population of DBG and FDBG

Four samples of DBG and FDBG were analyzed for measuring pH, lactobacillus and coliform bacteria counts, and the chemical composition. To determine the pH, 20 grams of each sample were transferred to a 250 mL beaker, and 200 mL of distilled water was added (Chiang *et al*., 2010). The pH was then measured using a pH meter (pH Meter NWKbinar pH, K-21, Landsberg, Germany). A 1-g sample of DBG and FDBG was used for the microbial population, as described by Ashayerizadeh *et al*. (2017). The composition of the samples was analyzed according to the standard AOAC (2005) procedures, including dry matter (DM) by oven-drying (method 930.15), ash, crude protein (CP) by Kjeldahl procedure (nitrogen × 6.25; method 990.02), ether extract (by Soxhlet; method 920.39), crude fiber (CF) by extraction in acid and alkali solution (method 978.10), neutral detergent fiber (NDF), and acid detergent fiber (ADF), based on the methods described in by van Soest *et al*. (1967). The BDG and FBDG ME were calculated according to the following formula: ME = (39.15 × DM) – (39.15 × ash) – (9.72 × CP) – (63.81 × CF) (NRC, 1994).

### Birds and diets

A total of 360 one-day-old male broiler chicks (Ross 308) were raised on a bedding of rice husk for 42 days. Birds had free access to water and feed throughout the experiment. The temperature, humidity, lighting programs, and ventilation were controlled according to the strain guidelines (Aviagen, 2018). The experimental treatments consisted of the following diets: 1) a corn and soybean meal-based diet (the control), 2) 10 % FDBG, 3) 10 % DBG, 4) 20 % FDBG, and 5) 20 % DBG. Each treatment had six replicates, each consisting of 12 birds. The chemical composition and amino acid (AA) values of feedstuffs were determined using Near Infrared Reflectance (NIR) technology. The composition of the diets during the starter (1-10 d), grower (11-24 d), and finisher (25-42 d) periods was based on the nutritional requirements of the Ross 308 (Table 2).

**Table 2:** Ingredients and chemical composition of the experimental diets.

Ingredients (%(	Starter (1–10 d)					Grower (11–24 d)					Finisher (25–42 d)				
	Control	DBG		FDBG		Control	DBG		FDBG		Control	DBG		FDBG	
		10 %	20 %	10 %	20 %		10 %	20 %	10 %	20 %		10 %	20 %	10 %	20 %
Corn	55.47	47.90	40.32	47.90	40.32	60.61	53.04	45.45	53.04	45.45	66.81	59.22	51.64	59.22	51.64
Soybean meal	35.47	31.60	27.74	31.60	27.74	30.83	26.96	23.10	26.96	23.10	24.99	21.12	17.25	21.12	17.25
DBG	0	10	0	10	0	0	10	0	10	0	0	10	0	10	0
FDBG	0	0	20	0	20	0	0	20	0	20	0	0	20	0	20
Corn gluten meal	3.00	3.00	3.00	3.00	3.00	3.00	3.00	3.00	3.00	3.00	3.00	3.00	3.00	3.00	3.00
Sunflower oil	1.20	2.62	4.03	2.62	4.03	1.20	2.62	4.03	2.62	4.03	1.20	2.62	4.03	2.62	4.03
Limestone	0.82	0.79	0.75	0.79	0.75	0.76	0.72	0.69	0.72	0.69	0.71	0.68	0.64	0.68	0.64
Dicalcium phosphate	2.15	2.18	2.22	2.18	2.22	1.89	1.92	1.96	1.92	1.96	1.69	1.72	1.76	1.72	1.76
Salt	0.29	0.29	0.29	0.29	0.29	0.28	0.28	0.28	0.28	0.28	0.25	0.25	0.25	0.25	0.25
Sodium bicarbonate	0.16	0.16	0.16	0.16	0.16	0.16	0.16	0.16	0.16	0.16	0.14	0.14	0.14	0.14	0.14
Vitamin premix^a^	0.25	0.25	0.25	0.25	0.25	0.25	0.25	0.25	0.25	0.25	0.25	0.25	0.25	0.25	0.25
Mineral premix^b^	0.25	0.25	0.25	0.25	0.25	0.25	0.25	0.25	0.25	0.25	0.25	0.25	0.25	0.25	0.25
DL-Met	0.36	0.35	0.34	0.35	0.34	0.30	0.29	0.28	0.29	0.28	0.26	0.25	0.25	0.25	0.25
L-Lys HCL	0.41	0.45	0.50	0.45	0.50	0.34	0.38	0.43	0.38	0.43	0.34	0.39	0.44	0.39	0.44
L-Thr	0.12	0.11	0.10	0.11	0.10	0.08	0.08	0.08	0.08	0.08	0.06	0.06	0.5	0.06	0.5
Coccidiostat	0.05	0.05	0.05	0.05	0.05	0.50	0.50	0.05	0.50	0.05	0.05	0.05	0.05	0.05	0.05
Analyzed chemical composition (%), except ME															
ME (Kcal/kg)	2860	2860	2860	2860	2860	2920	2920	2920	2920	2920	3000	3000	3000	3000	3000
Crude protein	21.93	21.93	21.93	21.93	21.93	20.27	20.27	20.27	20.27	20.27	18.28	18.28	18.28	18.28	18.28
Lys	1.37	1.37	1.37	1.37	1.37	1.22	1.22	1.22	1.22	1.22	1.09	1.09	1.09	1.09	1.09
Met	0.70	0.70	0.70	0.70	0.70	0.62	0.62	0.62	0.62	0.62	0.56	0.56	0.56	0.56	0.56
Met+Cys	1.03	1.03	1.03	1.03	1.03	0.93	0.93	0.93	0.93	0.93	0.85	0.85	0.85	0.85	0.85
Thr	0.92	0.92	0.92	0.92	0.92	0.83	0.83	0.83	0.83	0.83	0.73	0.73	0.73	0.73	0.73
Ca	0.92	0.92	0.92	0.92	0.92	0.82	0.82	0.82	0.82	0.82	0.74	0.74	0.74	0.74	0.74
Available P	0.46	0.46	0.46	0.46	0.46	0.41	0.41	0.41	0.41	0.41	0.37	0.37	0.37	0.37	0.37
Na	0.17	0.17	0.17	0.17	0.17	0.17	0.17	0.17	0.17	0.17	0.17	0.17	0.17	0.17	0.17

^a,b^The premixes provided the vitamin and minerals per kilogram of diet: vita A, 12000 IU; vita D3, 5000 IU; vit E, 80 IU; vita K3, 3.2 mg; vita B1, 3.2 mg; vita B2, 8.6 mg; niacin, 65 mg; pantothenic acid, 20 mg; vita B6, 4.3 mg; biotin, 0.22 mg; folic acid, 2.2 mg; vita B12, 0.017 mg; choline, 400 mg copper, 16 mg; iodine, 1.25 mg; iron, 20 mg; manganese, 120 mg; selenium, 0.3 mg; and zinc, 110 mg.

### Performance, morphology of ileum, and blood parameters

Birds were weighed at 1, 10, 24, and 42 days of age in pen-based groups, and the body weight gain was calculated for each rearing period. Feed intake was also measured during each rearing period. Then, FCR was calculated by dividing feed intake to body weight gain, taking into account the weights of mortality.

On day 42 of the experiment, one bird from each experimental unit with a body weight as close as to the mean body weight was selected for measuring blood parameters, carcass composition, ileal bacteria count, and intestinal morphometric indexes. Approximately 3 mL of blood was collected from the brachial vein, and the serum was separated by centrifuging at 1500 × *g* for 12 minutes (Mesgar *et al*., 2022). Total protein and albumin in the serum were assessed spectrophotometrically using standard kits (Pars Azmun Company, Karaj, Iran). The globulin content was calculated by subtracting albumin from total protein. The birds were then slaughtered to measure edible carcass, abdominal fat, ileal bacteria count, and ileal morphology. Bacterial populations in ileum contents were measured as previously described (Ashayerizadeh et al., 2017), with total anaerobic bacteria (TAB) by the plate count agar, LAB by modified de Man, Rogosa, Sharpe agar, and coliforms by violet red bile agar. For the histological evaluation of ileum villi, a 3-cm tissue sample was cut at about 5 cm distally away from the Meckel's diverticulum, rinsed with saline solution, and preserved in a 10 % formalin solution. Tissue samples were embedded in paraffin using a paraffin dispenser, and then 5 µm cross-sectional slices were prepared using a microtome (Leical RM 2145 model) and placed on slides. The slides were examined under a light microscope (Olympus CX41) at 100 magnification, and the height of the villi and the depth of crypts were measured using the Image J software (Bradley et al., 1994).

### Statistical analysis

The data related to the effect of the fermentation process on pH, bacteria population, and chemical composition of DBG and FDBG were analyzed based on a t-test procedure. Data related to the feeding periods of broiler chickens were analyzed in a completely randomized design. The statistical model was $y_{ij}=\mu +T_{i}+e_{ij}$, where y_ij_=observed response values, μ= overall mean, T_i_=effect of treatments, and e_ij_=random error. Significant differences among means were separated using the Tukey test at the 0.05 level of probability. An orthogonal polynomial contrast was used to test linear and quadratic trends in response to increasing levels of DBG and FDBG. All analyses were performed using SAS software (SAS, 2013).

## Results

### pH, bacterial count, and chemical composition of DBG and FDBG

The changes in pH, microbial population, and the chemical composition of DBG before and after fermentation are reported in Table 3. The fermentation process resulted in a 1.3 unit decrease in pH (*P* *<* *0.001*), which was associated with increased enumeration of lactic acid bacteria (LAB) in FDBG (8.23 cfu/g) compared to DBG (5.80 cfu/g) and eliminated coliform bacteria (*P* *<* *0.001*). The fermentation process caused a significant decrease in crude fiber (16.11 vs 15.53), NDF (56.37 vs 54.22), ADF (21.94 vs 19.88), and NFE (43.48 vs 42.46), with concomitant increases in ash (3.72 vs 4.45), calcium (0.20 vs 0.40), and phosphorus (0.20 vs 0.21). However, the fermentation process could not affect crude protein, dry matter, ether extract, and metabolizable energy of DBG (*P* *>* *0.05*).

**Table 3:** Effect of fermentation on pH value, bacteria population, and chemical composition of BDG and FBDG.

Items (% DM, unless mentioned)	BDG	FBDG	SED	*P-value*
pH	5.89	4.59	0.038	< 0.001
LAB (log_10_ cfu/g)	5.80	8.23	0.154	< 0.001
Coliforms (log_10_ cfu/g)	3.22	0	0.056	< 0.001
Dry matter (%)	90.18	90.24	0.073	0.482
Crude fiber	16.11	15.53	0.048	< 0.001
NDF	56.37	54.22	0.144	< 0.001
ADF	21.94	19.88	0.053	< 0.001
Ash	3.72	4.45	0.083	0.002
Calcium	0.20	0.40	0.003	< 0.001
Phosphorus	0.20	0.21	0.002	0.015
NFE	43.48	42.46	0.340	0.024
Ether extract	4.85	5.43	0.248	0.058
Crude protein	22.02	22.36	0.274	0.253
ME (kcal/kg)	2143	2150	4.706	0.429

BDG: dried brewer's grain; FBDG: Fermented dried brewer's grain; LAB: lactic acid bacteria; NDF: neutral detergent fiber; ADF: acid detergent fiber; NFE: nitrogen-free extract; SED: Standard error of the difference between means.

Data represent means of 4 samples per feed.

### Performance

The effects of dietary treatments on the growth performance of broiler chickens are presented in Table 4. Body weight gain of broiler chickens fed with 10 % FDBG was comparable to the control group in all feeding periods. Body weight gain of broiler chickens fed with 20 % FDBG or DBG was significantly lower than other treatments, especially in the starter period and the entire trial (*P* *=* *0.0001*). An orthogonal polynomial contrast showed that birds fed with FDBG had a relatively higher weight gain than those received DBG. A linear decline was observed in weight gain by an increase in DBG, while it was milder for FDBG (*P* < 0.01).

**Table 4:** The effect of FDBG and DBG levels on growth performance of broiler chickens.

Item	Treatments					SEM	ANOVA	DBG vs FDBG	P value			
	Contorl	FDBG 10 %	DBG 10 %	FDBG 20 %	DBG 20 %				DBG (Linear)	DBG (Quadratic)	FDBG (Linear)	FDBG (Quadratic)
Weight gain (g /day)												
1-10 d	13.11a	13.23 ^a^	11.52 ^ab^	11.18 ^b^	11.05 ^b^	0.41	0.0010	0.0948	0.0054	0.3263	0.0103	0.0766
11-24 d	39.71 ^a^	37.84 ^ab^	33.70 ^b^	34.62 ^ab^	33.15 ^b^	1.26	0.0040	0.0850	0.0054	0.1407	0.0075	0.6419
25-42 d	76.18 ^a^	71.07 ^ab^	69.62 ^ab^	68.63 ^b^	66.02 ^b^	1.63	0.0030	0.2410	0.0014	0.5272	0.0009	0.4143
1-42 d	49.01 ^a^	46.22 ^ab^	43.82 ^bc^	43.61 ^bc^	41.96 ^c^	0.92	0.0001	0.0667	0.0001	0.1809	0.0007	0.9367
Feed intake (g / day)												
1-10 d	23.32	23.47	22.98	20.83	21.66	0.72	0.0632	0.4243	0.1383	0.6020	0.0244	0.12161
11-24 d	71.16 ^b^	74.66 ^ab^	73.18 ^ab^	81.23 ^a^	76.85 ^ab^	2.26	0.0420	0.1699	0.0963	0.7690	0.0025	0.5315
25-42 d	148.52 ^c^	159.22 ^abc^	151.64^bc^	173.81^a^	166.65^ab^	4.07	0.0009	0.2188	0.0049	0.2314	0.0004	0.6960
1-42 d	92.36^c^	97.93 ^abc^	93.59 ^bc^	104.55 ^a^	100.25 ^ab^	1.89	0.0007	0.1572	0.0048	0.2090	0.0004	0.8283
Feed conversion ratio (g feed/g gain)												
1-10 d	1.79	1.81	1.99	1.79	1.97	0.07	0.1188	0.8629	0.0780	0.1789	0.9911	0.7883
11-24 d	1.80 ^b^	1.97 ^ab^	2.18 ^ab^	2.36 ^a^	2.35 ^a^	0.10	0.0023	0.0635	0.0018	0.3886	0.0005	0.3526
25-42 d	1.98 ^b^	2.23 ^b^	2.21 ^b^	2.63 ^a^	2.53 ^a^	0.07	<0.0001	0.0336	<0.0001	0.5001	<0.0001	0.5225
1-42 d	1.91 ^c^	2.13 ^c^	2.19 b^c^	2.50 ^a^	2.44 ^ab^	0.06	<0.0001	0.0219	<0.0001	0.8683	<0.0001	0.4141

DBG: dried brewer's grain; FDBG: fermented dried brewer's grain.

Means with different superscripts in each row are significantly different (*P* < 0.05).

SEM: Standard error of the means.

No significant difference was observed between dietary treatments for feed intake during the starter period (*P* *>* *0.05*). Meanwhile, in the grower, finisher, and entire period, the least feed consumption was observed in the control group, whereas the highest feed consumption was observed in birds fed with DBG or 20 % FDBG (*P* *<* *0.01*). An orthogonal polynomial contrast revealed that chickens fed FDBG had a relatively higher weight gain than those fed DBG (*P* *=* *0.0667*). A linear increasing trend was observed in feed intake by an increase in dietary level of DBG or FDBG (*P* *<* *0.001*).

Birds fed the control diet had a significantly lower FCR at all experimental periods, except for the starter, where no significant difference was observed between treatments. In contrast, greater FCR values were observed in birds fed with DBG or 20 % FDBG (*P* *<* *0.001*).

### Carcass characteristics and morphology of ileum

Dietary treatments had no significant effect on proportional carcass weights (*P* > 0.05, Table 5). Ileal villus height was significantly lower in broilers fed with 20 % DBG compared to the control treatment (*P* *=* *0.034*, Table 5), while no significant difference was observed between other treatments. No significant difference was observed between treatments with respect to crypt depth and the villus height to crypt depth ratio (*P* > 0.05, Table 5).

**Table 5:** Effects of FDBG and DBG levels on carcass characteristics (% of live body weight) and gut morphometric indices of broiler chickens.

Item	Carcass	Abdominal fat	Villus height (µm)	Crypt depth (µm)	Villus height/Crypt depth
Control	65.93	0.67	1280.2 ^a^	240.08	5.38
10 % FDBG	64.86	0.72	1101.5 ^ab^	246.89	4.50
10 % DBG	66.21	0.76	1167.5 ^ab^	255.68	4.62
20 % FDBG	65.27	0.70	995.6 ^ab^	230.95	4.24
20 % DBG	65.73	0.69	951.6 ^b^	208.65	4.61
SEM	0.86	0.02	75.38	13.93	0.303
*P- value*					
ANOVA	0.6840	0.1352	0.0344	0.1892	0.15
DBG vs FDBG	0.6372	0.0582	0.0011	0.1171	0.422
DBG (Linear)	0.4175	0.4189	0.0011	0.1171	0.101
DBG (Quadratic)	0.4922	0.0104	0.4788	0.0750	0.339
FDBG (Linear)	0.4106	0.3747	0.0285	0.6476	0.014
FDBG (Quadratic)	0.2878	0.2626	0.7256	0.5124	0.401

DBG: dried brewer's grain; FDBG: Fermented dried brewer's grain.

Means with different superscripts in each column are significantly different (*P* < 0.05).

SEM: Standard error of the means.

### Blood protein content

Table 6 indicates circulatory protein levels in broiler chickens fed different treatments. A significant increase was observed in serum concentration of total protein (*P* *=* *0.01*) and globulin (*P* *=* *0.0079*) in broilers fed with 10 % FDBG compared to the control group. The highest concentration of albumin was observed in broilers that received 20 % DBG, which was significantly greater than that in the control or 20 % FDBG groups (*P* *=* *0.017*). The lowest albumin to globulin ratio was observed in broilers on 10 % FDBG, which significantly differed from the control and 20 % DBG (*P* = 0.023).

**Table 6:** The effect of FDBG and DBG levels on the haematological parameters of broiler chickens.

Item	Total protein (g/dL)	Albumin (g/dL)	Globulin (g/dL)	Albumin / Globulin
Contorl	3.21 ^b^	1.18 ^b^	2.03 ^b^	0.58^a^
10 % FDBG	3.97 ^a^	1.24 ^ab^	2.73 ^a^	0.46 ^b^
10 % DBG	3.55 ^ab^	1.27 ^ab^	2.28 ^ab^	0.56 ^ab^
20 % FDBG	3.43 ^ab^	1.18 ^b^	2.25 ^ab^	0.53 ^ab^
20 % DBG	3.62 ^ab^	1.31 ^a^	2.31 ^ab^	0.57 ^a^
SEM	0.13	0.03	0.12	0.027
ANOVA	0.0100	0.0175	0.0079	0.023
DBG vs FDBG	0.5871	0.5209	0.4374	0.2341
DBG (Linear)	0.0456	0.0089	0.1152	0.9044
DBG (Quadratic)	0.5352	0.4222	0.4505	0.5617
FDBG (Linear)	0.2718	0.9502	0.2002	0.2050
FDBG (Quadratic)	0.0014	0.1731	0.0008	0.0050

Control: basal diet, DBG dried brewer's grain. FDBG: Fermented dried brewer's grain.

Means with different superscripts in each column are significantly different (*P* < 0.05).

SEM: Standard error of the means.

### Ileal bacteria counts

The effect of FDBG and DBG on bacteria population in the ileum of broiler chickens is presented in Table 7. There were insignificant differences between treatments for TAB, LAB, and coliform population in the ileum contents.

**Table 7:** The effect of FDBG and DBG levels on the ileal bacteria counts of broiler chickens (log_10_ cfu/g).

Item	TAB	LAB	Coliforms
Contorl	6.06	5.91	5.68
10 % FDBG	6.13	5.99	5.73
10 % DBG	6.05	5.90	5.65
20 % FDBG	6.13	5.98	5.72
20 % DBG	6.13	5.97	5.72
SEM	0.04	0.03	0.04
ANOVA	0.285	0.274	0.723
DBG vs FDBG	0.8288	0.9298	0.6998
DBG (Linear)	0.1239	0.2085	0.5850
DBG (Quadratic)	0.2010	0.3277	0.3351
FDBG (Linear)	0.3075	0.2487	0.6132
FDBG (Quadratic)	0.4506	0.3804	0.6517

Control: basal diet, DBG dried brewer's grain. FDBG: Fermented dried brewer's grain.

SEM: Standard error of the means: TAB: total anaerobic bacteria: LAB: lactic acid bacteria.

## Discussion

### Microbial and Chemical Composition of DBG

Aspergillus oryzae provides an aerobic environment for the growth of Bacillus subtilis by removing oxygen (Ashayerizadeh *et al*., 2017). Biological activities of Bacillus subtilis lead to the growth of LAB and increased lactic acid production, ultimately reducing the pH of the environment (Sugiharto and Ranjitkar, 2019). During the fermentation process, sugar compounds *(*D-fructose, d-mannitol, d-glucose, d-galactose, and maltose*)* are decreased as they are used for the growth of Bacillus subtilis. This results in an increase in the number of LAB and a decrease in pH (Tan et al., 2019). In line with the present experiment, it has been reported that the fermentation of fish waste (Shabani *et al*., 2021) and cottonseed meal (Jazi et al., 2017) caused an increase in LAB production with a concomitant decrease in pH. It has been reported that LAB increased from 7.5 to 8.2, while pH and coliform bacterial count were decreased after the fermentation process (Ranjitkar *et al*., 2016), which is in agreement with the present findings. Sugiharto and Ranjitkar (2019) also reported that fermentation increases LAB and acetic acid with a decrease in pH and Enterobacteriaceae.

A rise in the ether extract content of DBG has been reported following the fermentation process (Lunn *et al*., 2006), which is in line with the present experiment. Such an increase comes from the lipase activity of Bacillus subtilis bacteria during the fermentation process, which hydrolyzes lipids. In addition, Tan *et al*. (2019) demonstrated that increased ether extract content was linked to an increase in precursors of acetyl-CoA due to the glycolytic activity during fermentation. Lynch *et al*. (2016) reported that DBG has a high concentration of fiber (20- 25 % hemicellulose, 12- 25 % cellulose, and 10- 28 % lignin). The main component of hemicellulose (about 40 %) is arabinoxylans, and the most abundant monosaccharides in DBG are xylose, glucose, arabinose, rhamnose, and galactose (Forssell *et al*., 2008). Li *et al*. (2022) reported that LAB produces glucosidase enzymes, which leads to cellulose hydrolysis. The fermentation process also alters the monosaccharide composition of fibers. The type and complexity of glycosidic linkages, monosaccharide molecular arrangement, molecular weight, particle size, solubility, and physical properties influence fiber fermentation.

The fermentation process is associated with the occurrence of various microbial enzymes, such as cellulase and hemicellulase (Lincoln *et al*., 2017), which degrade the fiber content of DBG (Neylon et al., 2023). Therefore, the fermentation process reduces CF, NDF, and ADF contents. In line with this experiment, it has been reported that processing rapeseed meal (Ashayerizadeh *et al*., 2018) and cottonseed meal (Jazi *et al*., 2017) through fermentation increases ash content. An increase in ash may result from the reduction of crude fiber during the fermentation which shows the optimum fermentation period for DBG (Chikwendu *et al*., 2014). Tan *et al*. (2019) reported that amylase and cellulase enzymes produced by Bacillus subtilis during fermentation hydrolyze long-chain polysaccharides and convert starch molecules into simple sugars. An increase in calcium and phosphorus contents during fermentation could explain that the fermentation improves the bioavailability of minerals (Chikwendu *et al*., 2014). Microorganisms use carbon as the energy source during fermentation (Neylon et al., 2023).

### Growth performance

The results of this experiment showed that body weight was decreased by using DBG in the diet of broiler chickens, which is similar to the findings of Denstadli et al. (2010). Onifade and Babatunde (1998) reported that increasing the level of malt pomace in the diet (10 %, 15 % and 20 %) caused a decrease in body weight and an increase in feed consumption, which aligns with the present study. Similarly, Aghabeigi *et al*. (2013) showed that the substitution of malt pomace (0 %, 5 %, 10 %, 15 %, 25 %) for soybean meal negatively affected the performance of broiler chickens. Parpinelli et al. (2017) reported that the inclusion of 10 % DBG could be possible in the diet of broiler chickens without any adverse effect on performance. However, Abd El-Hack *et al*. (2019) stated that DBG can be used at dietary levels below 10 %. The restriction of DBG in the feed formulation can be attributed to the presence of NSPs, tannins and fibers (20-25 % hemicellulose, 12-25 % cellulose and 10-28 % lignin) (Lacassagne et al.*,* 1998; Lynch et al.*,* 2016; El-Hack *et al*., 2019). Alabi *et al*. (2014) used four enzyme cocktails (Roxazyme100, Nutrase150, Xyla, Grindazyme, at 350 ppm) in diets consisting of 25 % DBG. They found that the inclusion of enzymes improved growth performance of broiler chickens as opposed to the control without any enzyme supplement.

Adding enzymes to diets containing DBG is one way to reduce the level of NSPs and to increase the consumption of DBG through improving endogenous enzyme activity, digestion and nutrient absorption (Alabi, 2014). However, adding commercial enzymes to poultry feed has limitations due to the wide range of pH along the intestine with the favorable pH for enzyme activity (being between 4 and 6), the short digestion time in the proximal intestinal area (the main site of enzyme activity), and the probability of hydrolysis by endogenous proteolytic enzymes (Ravindran, 2013). Similar to the current experiment, Al-Khalaifah *et al*. (2020) used 5, 10, and 15 % of DBG with commercial enzymes, and the fermented DBG at levels of 5, 10, and 15 % without the enzyme. They reported that replacing corn-soybean based diet with 10 % FDBG or 5 % DBG plus enzyme resulted in a better growth and a higher economic efficiency of broilers chickens. Svihus *et al*. (1997) reported that the fermentation of barley reduced anti-nutritional compounds (ꞵ-glucans) by 62 %, and improved broiler chicken performance. The use of fermented DBG to improve performance can be attributed to the fermentation process, which increases the palatability and activity of ꞵ-glucanase, phytase, amylase, trypsin, lipase, and protease enzymes (Sugiharto and Ranjitkar, 2019). Al-Khalaifah *et al*. (2020) pointed out that the mechanism for improving digestion with fermented DBG in broiler chickens involves increasing the levels of nutrient-transporter genes (Pre-Stomach PGC and PGA5, Pancreas AMY2A, PNLIP, CELA1, and CCK; Duodenum CAT1, CAT2, GLUT1, GLUT2, and LAT1) involved in the digestion and transport of nutrients for better energy access and growth. Tan *et al*. (2019) also reported that fermenting DBG produces amino acids, increases unsaturated fatty acids, enhances antioxidant properties and phenolic content, and reduces NSPs and fiber. The use of fermented feed improves gut health and performance in broilers due to increased production of organic acids by LAB, which reduces pH through competitive exclusion, antagonistic activities, and bacteriocin production, thereby inhibiting pathogenic *E. coli, Salmonella*, coliform bacteria, and Enterobacteriaceae (Niba et al., 2009; Shabani *et al*., 2021). They stated that the increased production of short-chain fatty acids (Sugiharto and Ranjitkar., 2019; Vriesekoop *et al*., 2021) improved digestion and growth in broilers. Ashour *et al*. (2019) demonstrated that using DBG up to 9 % has no detrimental effect on growth performance in broilers.

### Carcass characteristics, bacteria counts and morphology of ileum

Similar to the current experiment, Kokol *et al*. (2012) reported that using 5, 10, and 15 % levels of untreated DBG had no significant effect on carcass characteristics of broilers. Nie *et al*. (2015) reported that feeding broilers with fermented cottonseed meal (containing LAB) reduced the relative weight of abdominal fat by modulating fatty acid oxidation and triglyceride hydrolysis. LAB reduces the activity of the acetyl-CoA carboxylase enzyme, which is responsible for fatty acid synthesis in the liver. A decrease in the activity of this enzyme results in reduced fatty acid production, limiting triglyceride storage in adipose tissue (Ashayerizadeh *et al*., 2018). Al-Khalaifah *et al*. (2020) reported that using fermented DBG in broiler diets improved carcass weight but had no significant effect on abdominal fat.

In the present experiment, the fermentation process resulted in a greater number of LAB and a lower count of coliforms of DBG. However, feeding FDBG could not alter the bacterial population in ileal contents. It was obviously expected that fermented feeds could increase LAB and decrease coliform counts as described by Jazi et al. (2017) and Ashayerizadeh et al. (2018). Similar to the current experiment, Ranjitkar et al. (2016) indicated that fermented feed had no influence on the diversity of the gut microbiota of broiler chickens. The effectiveness of fermented feeds depends on the balance of LAB, feed substrate and fermentation conditions capable of producing repeatable fermentation results (Niba *et al*., 2009). Hence, it seems that the inclusion of 10 to 20 % FDBG was not adequate to alter bacterial population in ileum contents in broiler chickens.

Broilers fed 20 % DBG had significantly shorter villus heights. It seems the increase of crude fiber in the treatment containing 20 % DBG led to reduce villus height, which consequently decreases the absorption of nutrients and slows down the growth (Sun *et al*., 2013). Mathlouthi *et al*. (2002) also reported that a decrease in villus length is associated with reduced intestinal absorption capacity and a smaller absorptive surface area available for nutrient uptake. Broilers fed the diet containing FDBG had morphometric indices similar to the control group. Improvements in morphometric indices in the small intestine of broilers by feeding fermented feeds have been discussed by Jazi *et al*. (2017). They reported three reasons, including the direct relationship between gastrointestinal microflora and the health of intestinal morphology, the decomposition of large proteins into small peptides during fermentation, and the reduction of anti-nutritional factors in the feed. Several studies indicated that the use of fermented fish waste silage (Shabani *et al*., 2021), fermented soybean meal (Feng *et al*., 2007), fermented rapeseed meal (Hu *et al*., 2016) and fermented flax meal (Sun *et al*., 2013) caused an increase in villus height and a decrease in intestinal crypt depth.

### Blood protein contents

While total protein and albumin concentrations reflect the metabolic status of protein, globulin concentration indicates the immune status in chickens (Lo *et al*., 2023). In the current experiment, FDBG at 10 % level caused the highest concentrations of total protein and globulin, which indicates nutrient priority toward weight gain (Zhu *et al*., 2023). Increased villus height in the treatment containing 10 % FDBG and the formation of small peptides during the fermentation process may have caused an elevation of blood globulin (Sugiharto and Ranjitkar, 2019). Such increase in villus height and blood globulin level was not observed in the treatment containing 20 % FDBG, which may be related to the greater content of crude fiber. A high level of globulin in a 10 % FDBG diet may indicate the re-direction of nutrients toward the production of antibodies and other proteins that are involved in boosting the immune system, such as cytokines and acute-phase proteins (Shamji *et al*., 2012). Filipovic *et al*. (2007) reported that the ratio of albumin to globulin is decreased in response to environmental or metabolic stresses, which generally occur due to increased levels of blood globulins and mobilization of the body's immune system. Zhu *et al*. (2023) reported that fermented feeds increase the concentration of serum total protein. Ashour *et al*. (2019) reported that the use of different levels of DBG has no significant effect on total protein and blood serum albumin. Al-Khalaifah *et al*. (2020) stated that no significant difference was observed in the concentration of total protein and globulin among consumers of the diet containing raw and fermented DBG, which agrees with the present results. Also Xu *et al*. (2012) reported that blood serum's total protein of broiler chickens was not affected by diets containing raw and fermented rapeseed meal.

## Conclusion

Based on the results of this study, the fermentation process was an effective method for improving the nutrient composition of DBG. While the use of DBG reduced the performance of broiler chickens, fermented DBG could improve the performance and gut morphology of broilers. DBG should be used at levels less than 10 % of broilers' diet, while FDBG may be used up to 10 %. Therefore, based on the positive effects of fermentation on nutrient compounds and the benefits of fermented DBG on the performance and villus height of broiler chickens, FDBG could partially replace with soybean meal regardless of its economical point.

## Declaration of competing interest

All authors have participated in (a) conception and design, or analysis and interpretation of the data; (b) drafting the article or revising it critically for important intellectual content; and (c) approval of the final version.

The authors have no conflicts of interest to declare.

This manuscript has not been submitted to, nor is under review at, another journal or other publishing venue.
